# A Comprehensive Spectroscopic and Computational Investigation to Probe the Interaction of Antineoplastic Drug Nordihydroguaiaretic Acid with Serum Albumins

**DOI:** 10.1371/journal.pone.0158833

**Published:** 2016-07-08

**Authors:** Saima Nusrat, Mohammad Khursheed Siddiqi, Masihuz Zaman, Nida Zaidi, Mohammad Rehan Ajmal, Parvez Alam, Atiyatul Qadeer, Ali Saber Abdelhameed, Rizwan Hasan Khan

**Affiliations:** 1 Interdisciplinary Biotechnology Unit, Aligarh Muslim University, Aligarh– 202002, India; 2 Department of Pharmaceutical Chemistry, College of Pharmacy, King Saud University, P.O. Box 2457, Riyadh, 11451, Saudi Arabia; University of Hyderabad, INDIA

## Abstract

Exogenous drugs that are used as antidote against chemotheray, inflammation or viral infection, gets absorbed and interacts reversibly to the major serum transport protein i.e. albumins, upon entering the circulatory system. To have a structural guideline in the rational drug designing and in the synthesis of drugs with greater efficacy, the binding mechanism of an antineoplastic and anti-inflammatory drug Nordihydroguaiaretic acid (NDGA) with human and bovine serum albumins (HSA & BSA) were examined by spectroscopic and computational methods. NDGA binds to site II of HSA with binding constant (K_b_) ~10^5^ M^-1^ and free energy (ΔG) ~ -7.5 kcal.mol^-1^. It also binds at site II of BSA but with lesser binding affinity (K_b_) ~10^5^ M^-1^ and ΔG ~ -6.5 kcal.mol^-1^. The negative value of ΔG, ΔH and ΔS for both the albumins at three different temperatures confirmed that the complex formation process between albumins and NDGA is spontaneous and exothermic. Furthermore, hydrogen bonds and hydrophobic interactions are the main forces involved in complex formation of NDGA with both the albumins as evaluated from fluorescence and molecular docking results. Binding of NDGA to both the albumins alter the conformation and causes minor change in the secondary structure of proteins as indicated by the CD spectra.

## 1. Introduction

The protein-drug interaction has been known as an extensive aspect towards the availability, efficacy and transport of drugs, for many years [1]. Drugs interact with circulating serum albumin of blood in a reversible manner and remarkably influence their absorption, apparent distribution volume, metabolism and excretion performance [2,3]. In addition, solubility of hydrophobic drugs in plasma improves on interaction with serum albumin which regulate their distribution to cells both *in-vivo* and *in-vitro* and consequently affect their disposition and efficacy [4]. Drug interaction with albumin also minimizes the rate of clearance and raises the plasma half-life of the drug. The binding affinities of various drugs and natural products to serum albumin decide their distribution and metabolism [5]. Thus, to study protein-drug interaction is essential to have an insight of the transport and distribution of drugs, and for the interpretation of the mechanism behind their action and pharmaceutical dynamics [6].

The polyphenolic compounds are notably considered for antitumor activity against human malignant tumors, such as melanoma, glioblastoma and adenocarcinoma of prostate and lungs [7], and thus considered as drug. One such naturally occurring polyphenol, Nordihydroguaiaretic acid (NDGA), is the dominant metabolite of Creosote bush *(Larrea tridentate)* which is used as remedy in the treatment of diversified form of diseases that include cardiovascular disorders, neurological disorders and different types of cancer [8,9]. It has been considered since decades due to its advancement as antineoplastic, antiviral and anti-inflammatory characteristic attributes [8,10]. NDGA acquires a broad spectrum of biological properties and encompasses o-dihydroxy (catechol) group; bearing four hydroxyl groups of two phenols on the edges and hydrocarbon at the centre. Further, it is identified as a strong antioxidant and an *in-vitro* scavenger of reactive oxygen species (ROS), with distinct valuable health effects [11,12]. NDGA has also been acknowledged to have potent anti-amyloidogenic and fibril disaggregating properties [13,14,15]. Being a lipoxygenase inhibitor NDGA has been shown to prevent the toxicity of Aβ on rat hippocampal neurons [14]. It also obstructs the tyrosine kinase behaviour of the IGF-1 receptor (IGF-1R) and the HER2 receptor in breast cancer cells [16]. Thus, the study of NDGA interaction with serum albumins has biological relevance. In the present study, an attempt has been made to understand and evaluate the binding affinity and mechanism of NDGA interaction with human serum albumin (HSA) and bovine serum albumin (BSA).

HSA comprises of 585 amino acid residues and possess three homologous domains I-III [17]. Each domain comprises of two sub domains (A and B) [2,18]. The sub domain IIA possess Sudlow's site I whereas Sudlow's site II is located in sub domain IIIA [19,20]. HSA and BSA have 76% of sequence homology and homologous disulphide bond arrangements [21]; however number of tryptophan residues differ (two in BSA whereas single in HSA). BSA possess three homologous domains (I-III), same sub domains and similar binding site as that of HSA but possess 583 amino acid residues [22].

In the present study, binding energetics of NDGA and serum albumins (HSA and BSA) were evaluated by the steady state fluorescence spectroscopic technique. Determination of binding site was performed by displacement studies. The conformational alteration was observed by CD spectroscopy and the size of NDGA-albumin complex were determined by DLS measurements. Recognition of the amino acid residues involved in interaction of NDGA and albumins were done by molecular docking studies.

## 2. Materials and Methods

### 2.1. Materials

Essentially globulin and fatty acid free Human serum albumin (A1887), Bovine serum albumin (A7030), Nordihydroguaiaretic acid (N5023), Warfarin (A2250) and Phenylbutazone (P8386) were procured from Sigma Aldrich. Diazepam was obtained from Ranbaxy Laboratories Ltd. All other reagents were of analytical grade. Double distilled water free from any fluorescent contaminants was used.

### 2.2. Preparation of Solutions

All experiments were carried out in 20 mM sodium phosphate buffer (pH 7.4). The stock solution of proteins (10 mg/ml) were also prepared in 20 mM sodium phosphate buffer pH 7.4 and dialyzed in the same buffer. The concentration of proteins were determined spectrophotometrically using extinction coefficient $E_{280\;\mathrm{nm}}^{1\%}$ = 5.3 M^-1^.cm^-1^ and 6.5 M^-1^.cm^-1^ (for HSA and BSA, respectively) on Perkin Elmer Lambda 25 spectrophotometer. NDGA (2 mg/ml) was prepared in ethanol which was diluted in sodium phosphate buffer pH 7.4, for further use.

### 2.3. Steady State Fluorescence Quenching Measurements

Quenching may occur due to different molecular interactions *viz*. ground-state complex formation, collisional quenching, excited state reactions, molecular rearrangements and energy transfer. Quenching is classified as either dynamic or static quenching [23]. Dynamic quenching results from the collisional encounter between the fluorophore and the quencher whereas static quenching arises due to the ground state complex formation between the fluorophore and the quencher. Dynamic and static quenching can be differentiated by their dependence on temperature and viscosity. Fluorescence emission spectra were recorded on a Schimadzu 5301 PC fluorescence spectrophotometer equipped with a water circulator (JulaboEyela). The excitation and emission slit width were set at 3 nm and 5 nm. Fluorescence measurements of HSA & BSA (5 μM each) solutions were taken during separate titration with NDGA (0–50 μM) from 1:0 to 1:10 molar ratio of protein to drug (at an increments of 5 μM), in a dual-path length fluorescence cuvette (10 × 3.5 mm). The experiment was carried out at three different temperatures (298 K, 303 K and 310 K) in order to monitor the temperature dependence of serum albumin (HSA & BSA) and NDGA interaction. Excitation wavelength of 295 nm was selected to avoid possibility of fluorescence emission by tyrosine residues [24]. The emission spectra were recorded in the wavelength range of 300–450 nm. Respective blanks were subtracted from the spectra. The data obtained were analyzed according to linear Stern-Volmer equation [25]:

$$\frac{F_{o}}{F}=K_{sv}[Q]+1=k_{q}\tau_{o}+1$$ (1)

where, *F*_*o*_ and *F* are the fluorescence intensities in the absence and presence of NDGA (quencher), K_sv_ is the Stern–Volmer quenching constant (measuring the efficiency of quenching), *k*_*q*_ is the bimolecular rate constant of the quenching reaction and τ_o_ is the average integral fluorescence life time of tryptophan which is ~10^−9^ s [26]. For the quenching process, the binding constant and the number of binding site were obtained by modified Stern-Volmer equation [26]:

$$\log\;(\frac{F_{o}}{F}-1)=\log\;k_{b}+nlog[Q]$$ (2)

where, *K*_*b*_ is the binding constant, *n* is the number of binding sites, [*Q*] is the concentration of quencher *i*.*e*. NDGA. The change in free energy *(ΔG°)* was calculated from Gibbs-Helmholtz equation (Eq 3) whereas the change in enthalpy *(ΔH°)* and entropy *(ΔS°)* at different temperatures were analyzed from the Van’t Hoff equation (Eq 4):

$$\Delta G{}^{\circ}=-RTlnK_{b}$$ (3)

$$lnK_{b}=\frac{-\Delta H}{RT}+\frac{\Delta S}{R}$$ (4)

where, *R* (1.987 cal.mol^-1^.K^-1^) is gas constant and *T* is the absolute temperature (K).

The synchronous fluorescence spectra were recorded when Δλ (difference between the excitation and emission wavelength) is 60 nm (for tryptophan) and 15 nm (for tyrosine) for HSA and BSA (5 μM each) in the absence and presence of 1:10 molar ratio of protein to NDGA (0–50 μM) over a wavelength range of 280–400 nm.

### 2.4. UV-Visible Spectroscopic Measurements

Absorption measurements were performed at 37°C on Perkin-Elmer Lambda 25 double beam UV–vis spectrophotometer attached with a peltier temperature programmer-1 (PTP–1). A fixed concentration of HSA and BSA (10 μM each) was titrated with NDGA (0–100 μM) from 1:0 to 1:10 molar ratio of protein to drug and the respective blanks were subtracted.

### 2.5. Fluorescence Resonance Energy Transfer (FRET) Measurements

The fluorescence spectra of HSA & BSA (5 μM each) and absorption the spectra of NDGA (5 μM) in the wavelength range of 300–400 nm were scanned at 25°C as mentioned above. By Forster’s theory, the efficiency of energy transfer (E) is calculated using the following equation: [27]

$$E=1-\frac{F}{F_{0}}=\frac{R_{0}^{6}}{R_{0}^{6}+r^{6}}$$ (5)

where, *F*_*o*_ and *F* are the fluorescence intensities of HSA and BSA in the absence and presence of NDGA respectively, *r* is the distance between the donor and the acceptor molecules and *R*_o_ is the critical distance at which transfer efficiency is 50% which can be calculated from the following equation:

$$R_{0}^{6}=8.79\times 10^{-25}K^{2}n^{-4}\emptyset J$$ (6)

where, *K*^*2*^ is the orientation factor related to the geometry of the donor and acceptor of dipoles, *n* is the refractive index of the medium, φ is the fluorescence quantum yield of the donor in the absence of acceptor, and *J* expresses the degree of spectral overlap between the donor emission and the acceptor absorption which can be evaluated by integrating the overlap spectral area in the wavelength range of 300–400 nm, from the following equation:

$$J=\int_{0}^{\infty }\frac{F(\lambda )\varepsilon \lambda^{4}d\lambda }{\int_{0}^{\infty }F(\lambda )d\lambda }$$ (7)

where, *F(λ)* is the fluorescence intensity of the donor at wavelength range *λ* which is dimensionless, and *ε(λ)* is the molar absorptivity (extinction coefficient) of the acceptor at wavelength *λ* in M^−1^.cm^-1^. The values for *K*^2^, φ and *n* were taken as 2/3, 0.15 and 1.336, respectively (for HSA as well as for BSA) [28].

### 2.6. Circular Dichroic Measurements

The far-UV and near-UV CD spectra of HSA and BSA in the absence and presence of NDGA were obtained using JASCO-J815 spectropolarimeter equipped with a Peltier-type temperature controller. Calibration of the instrument was performed with d-10-camphorsulfonic acid. All the CD measurements were done at 25°C. Spectra were obtained with 50 nm/min scan speed, 0.1 nm data pitch and a response time of 2 s. Each spectrum obtained was the average of 2 scans [29]. The path length of the cells were 0.1 cm for far-UV CD (190–250 nm) and 1 cm for near-UV CD (250–300 nm) measurements. The HSA and BSA concentration for far and near-UV CD measurements were taken as 5 μM and 10 μM. The percent of secondary structure was estimated by using online K_2_D software.

### 2.7. Dynamic Light Scattering (DLS) Measurements

DLS measurements were performed at 830 nm on a DynaPro-TC-04 dynamic light scattering equipment (Protein Solutions, Wyatt Technology, Santa Barbara, CA) attached with a temperature-controlled micro sampler. HSA and BSA (2 mg/ml each) were incubated with NDGA (25 μM and 50 μM) in the protein to drug molar ratio of 1:0, 1:5 and 1:10 for 8 hours. Further, the samples were spun at 10,000 rpm for 10 min and then filtered through 0.22 μm and 0.02 μm Whatman syringe filters into a 12 μl quartz cuvette [30]. For each experiment, 20 measurements were taken. Mean hydrodynamic radius (*R*_*h*_) and polydispersity were evaluated by using Dynamics 6.10.0.10 software at optimized resolution [31]. The *R*_*h*_ was measured on the basis of an autocorrelation analysis of scattered light intensity data depending on translation diffusion coefficient by Stoke’s-Einstein relationship:

$$R_{h}=\frac{kT}{6\pi \eta D}$$ (8)

where, *R*_*h*_ is the hydrodynamic radius, *k* is Boltzmann constant, *T* is temperature, *η* is the viscosity of water and *D* is diffusion coefficient [32].

### 2.8. Molecular Docking

The crystal structure of HSA (PDB id: 1AO6) and of BSA (PDB id: 4F5S) were taken from Brookhaven Protein Data Bank and the 3D structure of NDGA (CID: 4534) was obtained from PubChem. The docking studies were performed by auto dock 4.2.0 software [33]. Lamarckian genetic algorithm (LGA) implemented with an adaptive local method search was applied to rule out the possible conformation of NDGA that binds to the protein. Hydrogen atoms and water molecules were eliminated and then, partial Kollman charges were designated to the proteins (HSA and BSA). The proteins were set to be rigid and all the torsional bonds were taken as being free during the docking process. The solvent molecules were not considered during docking. To reveal the binding site of NDGA on HSA and BSA, blind docking were performed and the grid size was set to be 126, 126 and 126 along the X, Y and Z axes with 0.564 A° grid spacing. Auto dock parameters were used with 150 as GA population size and 2,500,000 as maximum number of energy evolutions. The ten best solutions based on docking score were retained for further investigations. Discovery studio 3.5 was used to visualize and recognize the residues involved in the binding process.

### 2.9. Statistical Analysis

Results were shown as the means and the standard deviation (S.D.) values, with n = 3 as the number of independent experiments.

## 3. Results and Discussion

### 3.1. NDGA Induced Fluorescence Quenching of HSA and BSA

**Fig 1:**
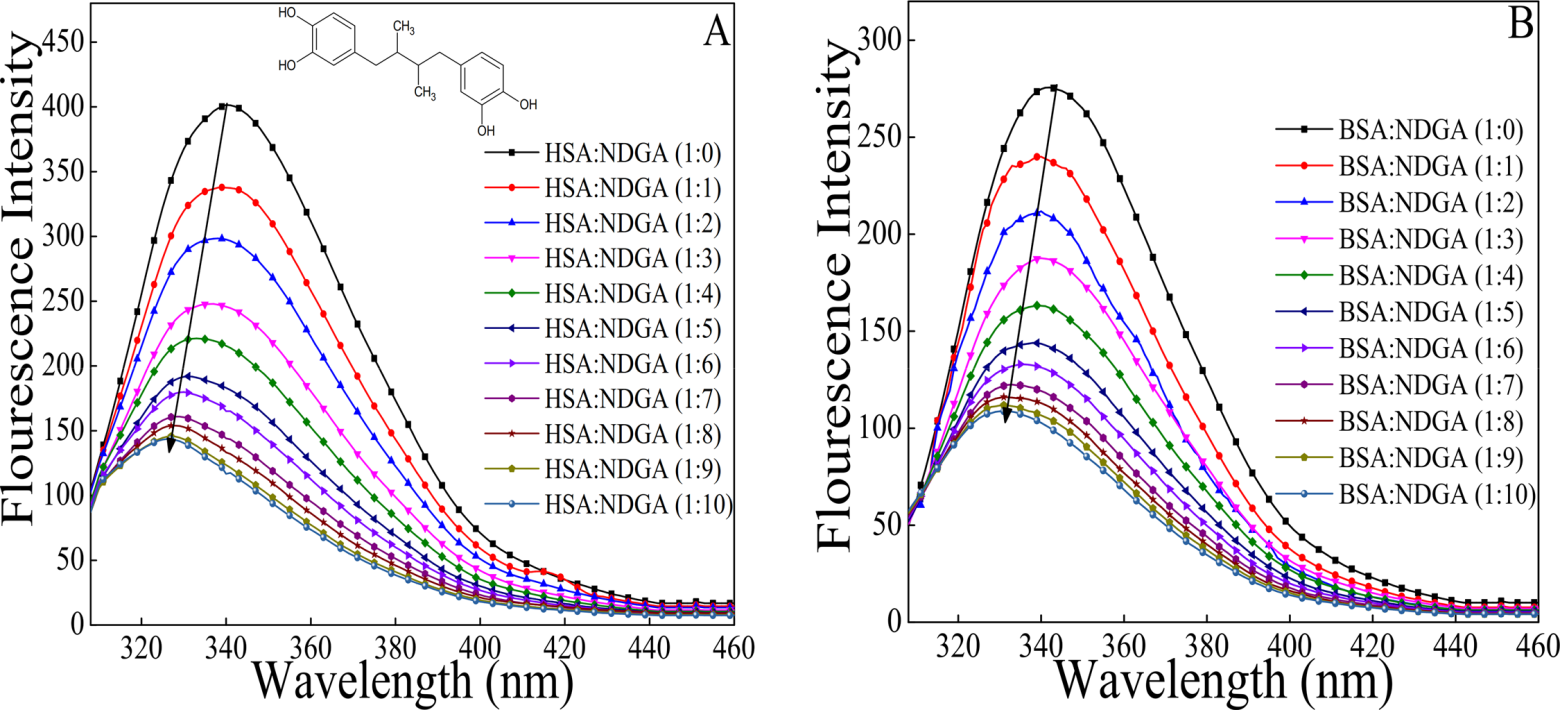
Emission spectra of albumins in the absence and presence of increasing concentration ofNDGA, from 1:0 to 1:10 molar ratio of albumins to NDGA. (A) HSA (B) BSA.

**Fig 2:**
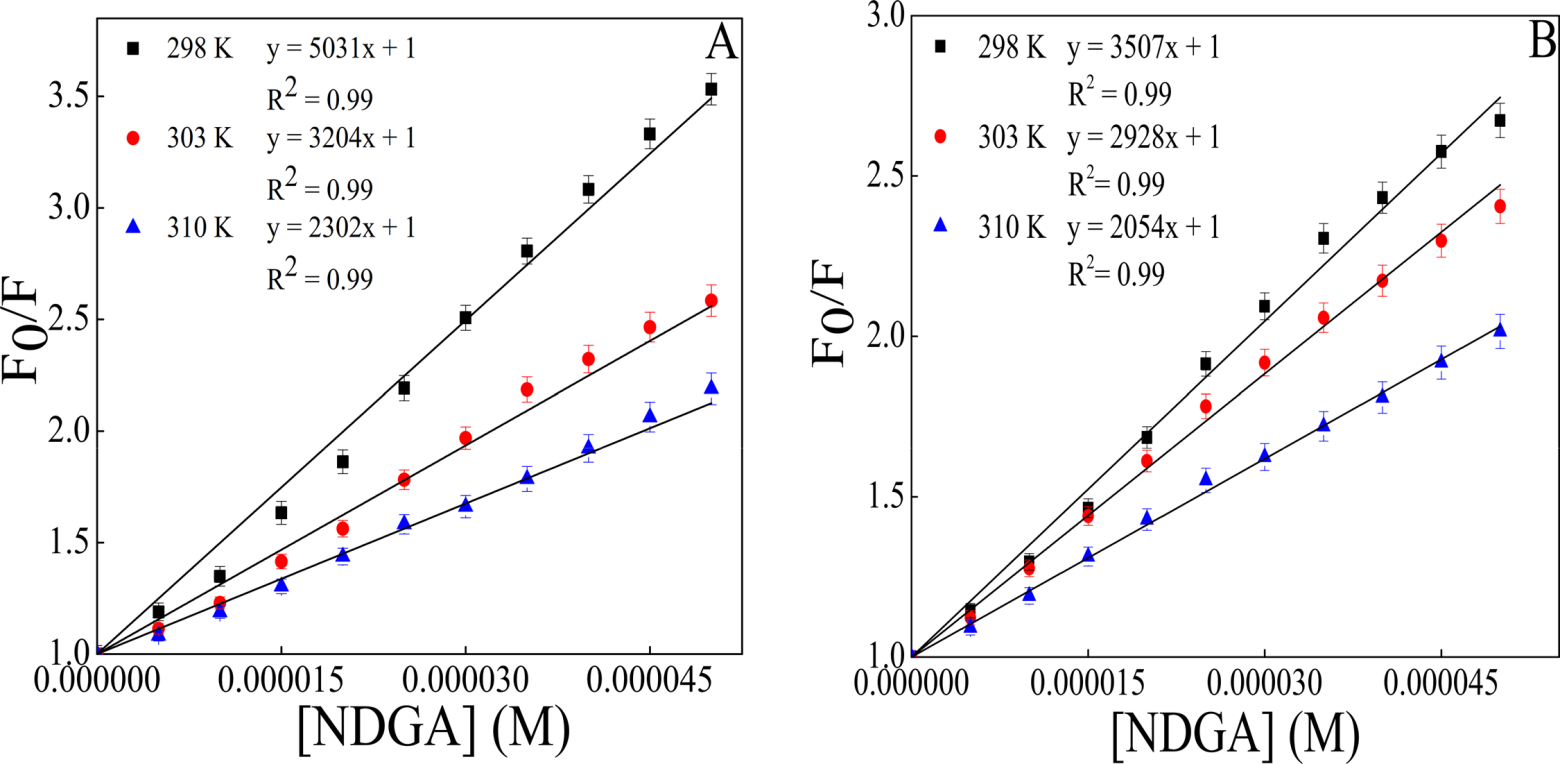
Stern-Volmer plot between Fo/F and [NDGA] for albumins–NDGA interaction. (A) for HSA–NDGA and (B) for BSA–NDGA at 298 K, 303 K and 310 K.

Fluorescence intensity measurement is a sensitive tool to probe the protein conformation and structure when microenvironment around fluorophore (Tyr, Trp and Phe) gets altered [34].The drug (NDGA) molecule quenches the fluorescence intensity of the albumins upto the ratio where it gets almost constant (from 1:0 to 1:10 molar ratio of protein to drug), due to its interaction with the fluorophores present in the proteins. Strikingly, intrinsic fluorescence property of protein is attributed due to the presence of aromatic amino acid residues viz, Trp, Tyr and Phe[35,36]. As shown in (Fig 1A and 1B) NDGA molecule quenched the fluorescence intensity of Trp in both the proteins. The increase in the concentration of NDGA caused decrease in the fluorescence intensity of HSA and BSA. However, addition of NDGA to proteins beyond 1:10 molar ratio of protein to NDGA does not caused any further decrement in the fluorescence intensity. This decrease in fluorescence intensities was accompanied by a blue shift of 14 nm and 10 nm for HSA and BSA, respectively. The decrement in fluorescence intensity with blue shift signifies that the interaction of NDGA with proteins resulted in alteration in microenvironment around tryptophan residue and moved it to a more hydrophobic environment [37,38]. The binding parameters obtained from fluorescence measurements according to Stern-Volmer equation are summarized in Table 1 (Fig 2A and 2B). Diffusion coefficient increases with increment in temperature resulting in higher dynamic quenching constant value. In contrast, poorly bound complexes gets destabilized at higher temperature and therefore lowers the static quenching constant value [26]. As can be seen from Table 1 that K_sv_ value decreases with increasing temperature that reflects the static nature of quenching process rather than dynamic quenching [39]. The value of *k*_*q*_ were found to be 1000 times greater than the maximum scatter collision quenching constant of various quenchers with biopolymer (2.0 × 10^10^ M^-1^.s^-1^) which also strongly implies that fluorescence quenching of serum albumins (HSA & BSA) by NDGA occurred through static quenching mechanism [40].

**Table 1:** Binding and thermodynamic parameters of Albumin-NDGA at different temperatures obtained from fluorescence quenching experiments^a^.

Protein	Temp (K)	n	K_SV_ (M^-1^)	k_q_(M^-1^ s^-1^)	K_b_ (M^-1^)	ΔH (kcal.mol^-1^)	ΔG (kcal.mol^-1^)	ΔS (cal.mol^-1^.K^1^)
**HSA**	298	1.1±0.02	(5.03±0.06) x10^4^	(5.03±0.06)x10^13^	(3.14±0.10)x10^5^		-7.46±0.04	-8.31±0.25
	303	1.1±0.01	(3.20±0.05) x10^4^	(3.20±0.05)x10^13^	(2.22±0.17)x10^5^	-15.806±0.06	-7.38±0.03	-8.45±0.30
	310	1.1±0.02	(2.30±0.07) x10^4^	(2.30±0.07)x10^13^	(1.12±0.12)x10^5^		-7.13±0.06	-8.65±0.17
**BSA**	298	1.1±0.03	(3.50±0.02) x10^4^	(3.50±0.02)x10^13^	(1.00±0.08)x10^5^		-6.79±0.05	-10.34±0.2
	303	1.0±0.04	(2.92±0.07) x10^4^	(2.92±0.07)x10^13^	(5.57±0.09)x10^4^	-17.115±0.04	-6.55±0.03	-10.51±0.3
	310	1.0±0.02	(2.05±0.07) x10^4^	(2.05±0.07)x10^13^	(3.22±0.12)x10^4^		-6.37±0.03	-10.75±0.1

^**a**^ R^2^ for all values ranges from 0.98 to 0.99

**Fig 3:**
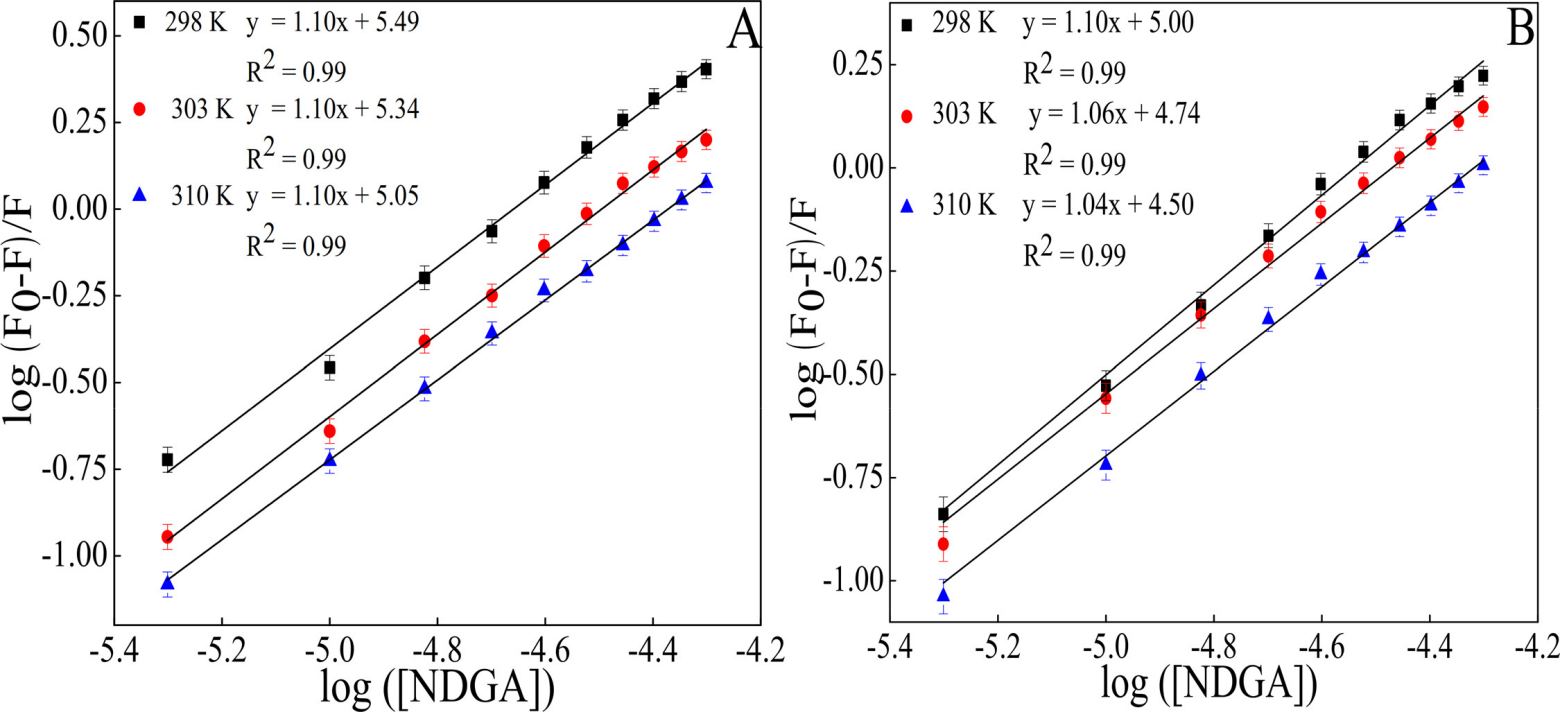
Plot between log [(Fo/F)-1] and log [NDGA] for (A) HSA-NDGA and (B) BSA-NDGA interaction at 298 K, 303 K and 310 K. Results are mean of threeindependent experiments (n = 3) and the error bars show the standard deviation

The K_b_ values obtained from the y-axis intercept of modified Stern-Volmer plot (Fig 3A and 3B), decreases with increase in temperature which suggests that higher temperature leads to the formation of less stable complex formation. The value of n is almost equal to unity demonstrating that there is one independent class of binding site for NDGA on both HSA as well as BSA.

### 3.2. Estimation of Thermodynamic Parameters

**Fig 4:**
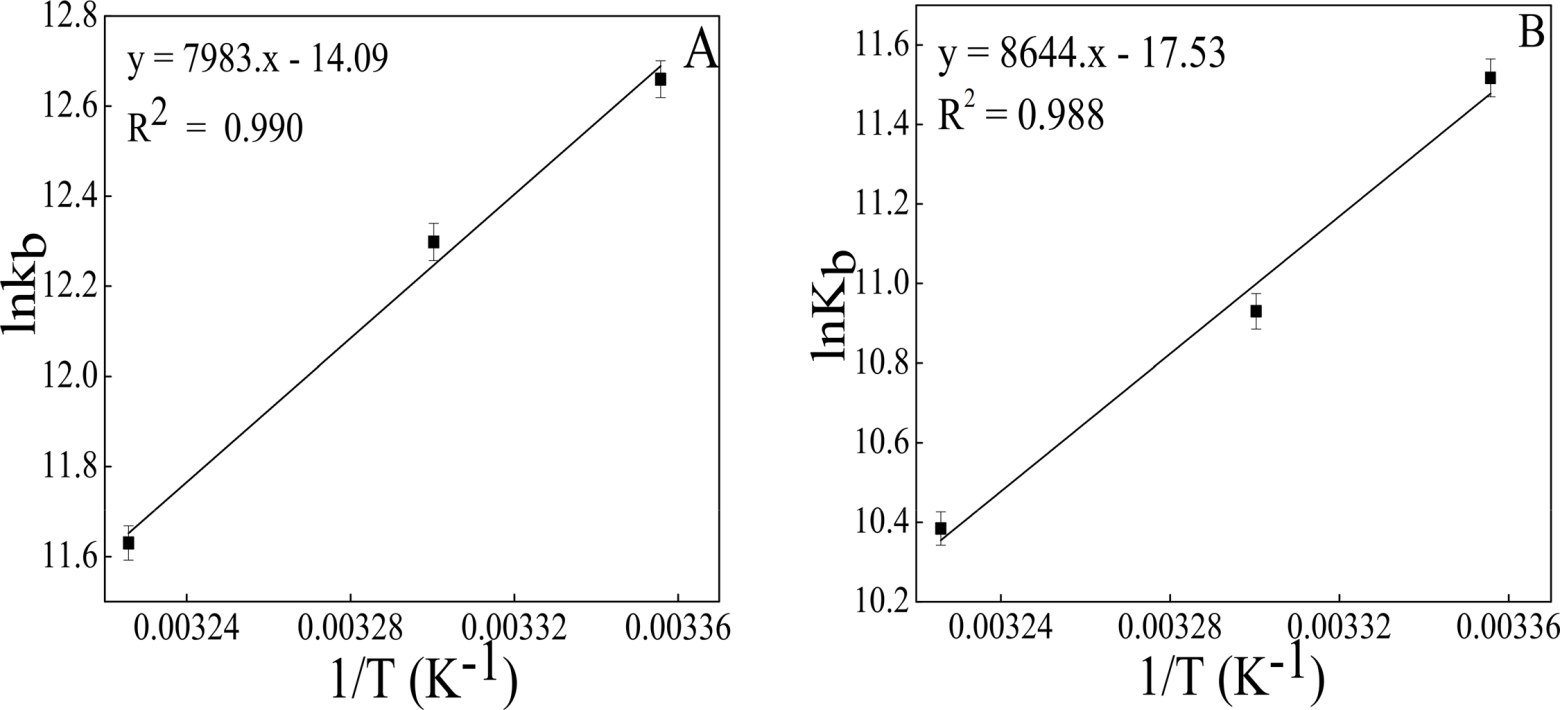
Van’t Hoff plot for temperature dependence of K_b_. Obtained from fluorescence quenching of albumins by NDGA at 298 K, 303 K and 310 K. Results are mean of threeindependent experiments (n = 3) and the error bars show the standard deviation

Evaluation of thermodynamic parameters is significant as it provides information regarding the binding forces involved during protein-drug interaction. The enthalpy change (ΔH) is calculated from the slope of the van’t Hoff plot as shown in Fig 4 whereas the entropy change (ΔS) is calculated from the intercept (Fig 4). The binding energetics obtained from van’t Hoff plot and Gibbs-Helmholtz equations are summarized in Table 1. The negative ΔG value indicates that NDGA interacts with albumins (HSA and BSA) in a spontaneous manner and the negative value of ΔH signifies the interaction process to be exothermic. According to the rules summarized by Ross and Subramanian [41], the negative value of ΔS and ΔH dictates that hydrogen bond plays a crucial role in Albumin-NDGA (HSA and BSA) complex formation. The role of hydrogen bond can be justified by the structure of NDGA which possess four hydroxyl groups that may contribute to the interaction of NDGA with the aromatic amino acid residues present in HSA and BSA. Significantly higher value of ΔH rules out the possibility of ionic forces as these forces are characterized by a ΔH≈0 [41].

### 3.3. Probing Binding Site using Site Markers

To get an insight into the binding site of NDGA on HSA and BSA, fluorescence displacement experiment was performed through a basic approach of competitive binding between NDGA and site specific markers viz. warfarin (WAR), phenylbutazone (PBZ) for site I and diazepam (DIA) for site II [42,43]. Albumins (HSA and BSA) and marker concentration were kept constant in the molar ratio of 1:1 and NDGA (0–50 μM) was used from 1:0 to 1:10 molar ratio of protein to drug. The drugs usually bind HSA and BSA at two major binding sites, Sudlow’s site I and site II, that are located in the specialized cavities in subdomains IIA and IIIA, respectively [44]. For the determination of the binding site of NDGA on HSA and BSA the values of K_sv_ in the absence and presence of markers (WAR, PBZ and DIA) were calculated by the Stern-Volmer equation and listed in Table 2. The value of K_sv_ in case of HSA as well as BSA decreased in the presence of site marker, DIA (Sudlow site II marker), PBZ and WAR (Sudlow site I markers) indicating the competition between the drug (NDGA) and the site markers for the same binding site. But the value of K_sv_was found to decrease by ten timesin the presence of DIA while the decrease in K_sv_ in the presence of PBZ and WAR was not much smaller but was significant thus the binding of NDGA to site I cannot be neglected (Table 2). This confirms Sudlow’s site II as the primary binding site and Sudlow’s site I as the secondary binding site of NDGA on HSA as well as on BSA [45,46].

**Table 2:** Effect of site markers upon NDGA binding to HSA and BSA.

Protein	K_sv_without site marker	R^2^	K_sv_with PBZ	R^2^	K_sv_with WAR	R^2^	K_sv_ with Dia	R^2^
**HSA**	5.03x10^4^	0.99	1.20x10^4^	0.99	1.7x10^4^	0.99	6.3x10^3^	0.99
**BSA**	3.50x10^4^	0.99	1.02x10^4^	0.99	1.8x10^4^	0.99	6.4x10^3^	0.99

### 3.4. Effect of NDGA on HSA & BSA Absorption Spectra

**Fig 5:**
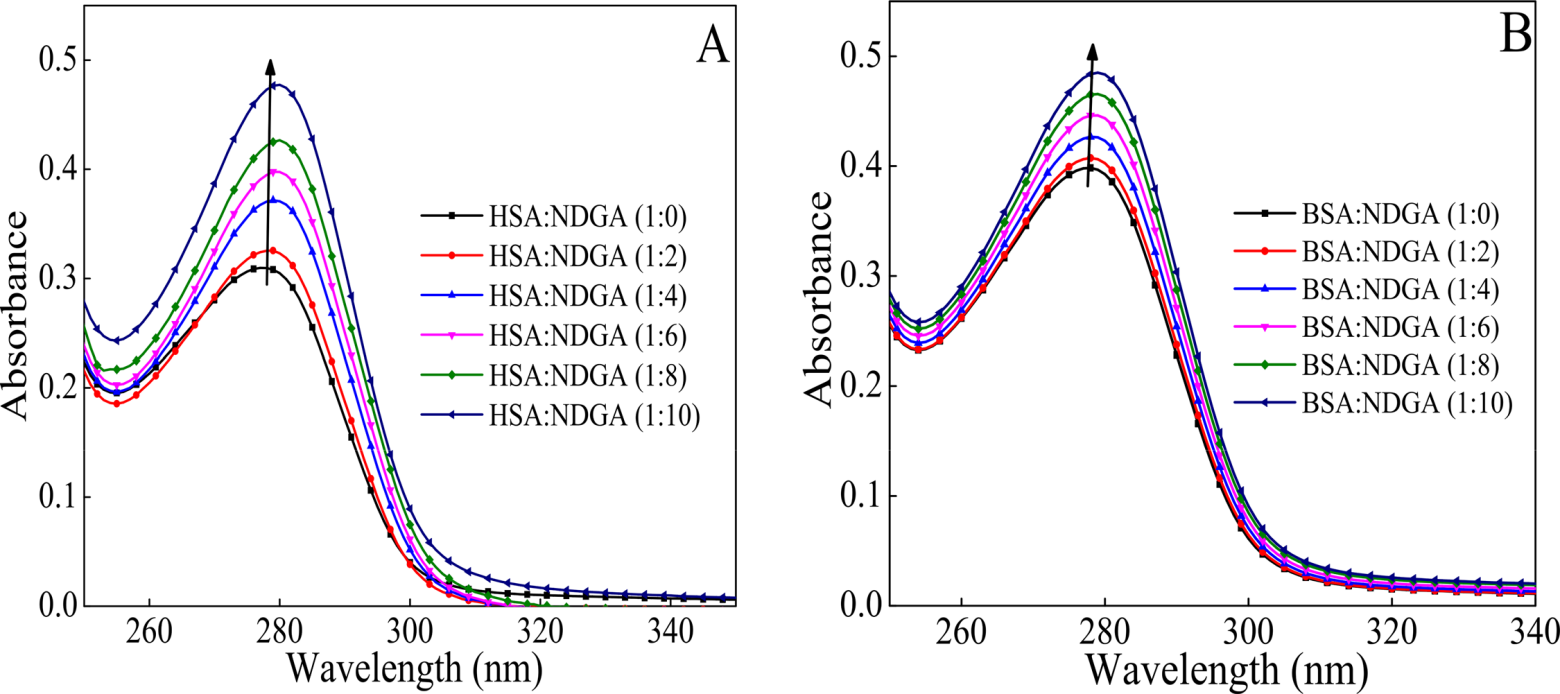
Absorption spectra of HSA (A) and BSA (B) gradually titrated with NDGA upto 1:10 molar ratio of albumins to NDGA, at 37°C.

UV-visible spectra of albumins give bands in the wavelength range of 250–300 nm which is a cumulative absorption of three aromatic amino acid residues viz. tryptophan, tyrosine and phenylalanine. Fig 5A and 5B shows the absorption spectra of HSA and BSA in the absence and presence of 1:0, 1:2, 1:4, 1:6, 1:8 and 1:10 molar ratio of protein to NDGA. It is evident from the graph obtained that NDGA interaction with HSA and BSA results in increase in absorbance around 280 nm which indicates the complex formation between NDGA and albumins (HSA and BSA) and thus resulted into the increase in maximal absorption peak (hyperchromic shift) [47].

### 3.5. Conformational Investigation

**Fig 6:**
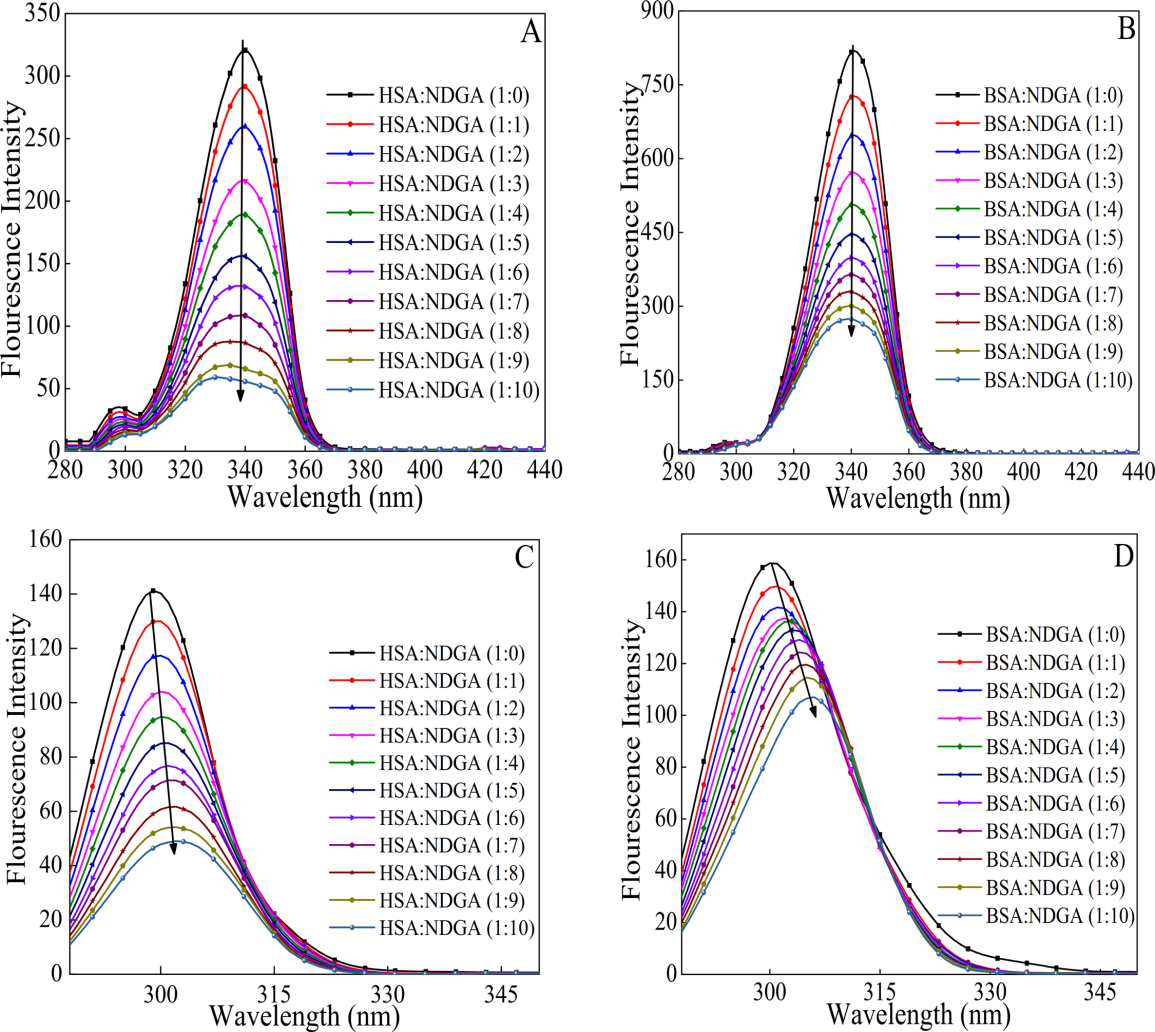
Synchronous fluorescence spectrum of HSA-NDGA (A) and BSA-NDGA (B) at Δλ = 60 nm and HSA-NDGA (C) and BSA-NDGA (D) at Δλ = 15 nm.

Synchronous fluorescence spectroscopy provides information about the change in microenvironment around the fluorophore (Trp and Tyr) present in the protein upon binding with the drug [48]. When the Δλ is 60 nm and 15 nm, synchronous fluorescence spectroscopy describes about the perturbation in microenvironment in the vicinity of Trp and Tyr residues, respectively. Fig 6A and 6C shows the synchronous fluorescence spectra of HSA when the Δλ was 60 nm and 15 nm, in the absence and presence of increasing protein to drug molar ratio of NDGA (from 1:0 to 1:10). Also, Fig 6B and 6D shows the synchronous fluorescence spectra of BSA, when the Δλ was 60 nm and 15 nm, in the absence and presence of increasing protein to drug molar ratio of NDGA (from 1:0 to 1:10). A decrease in fluorescence intensity with no shift in λ_max_ was observed when Δλ = 60 nm, indicating no change in the microenvironment around Trp, for HSA as well as for BSA, as shown in Fig 6A and 6B. In contrast, when Δλ = 15 nm, a red shift of 3 nm and 6 nm for HSA-NDGA and BSA-NDGA was observed as shown in Fig 6C and 6D. It shows that NDGA binding to HSA and BSA bring the Tyr residue to a hydrophilic environment.

### 3.6. Steady State Fluorescence Resonance Energy Transfer (FRET)

**Fig 7:**
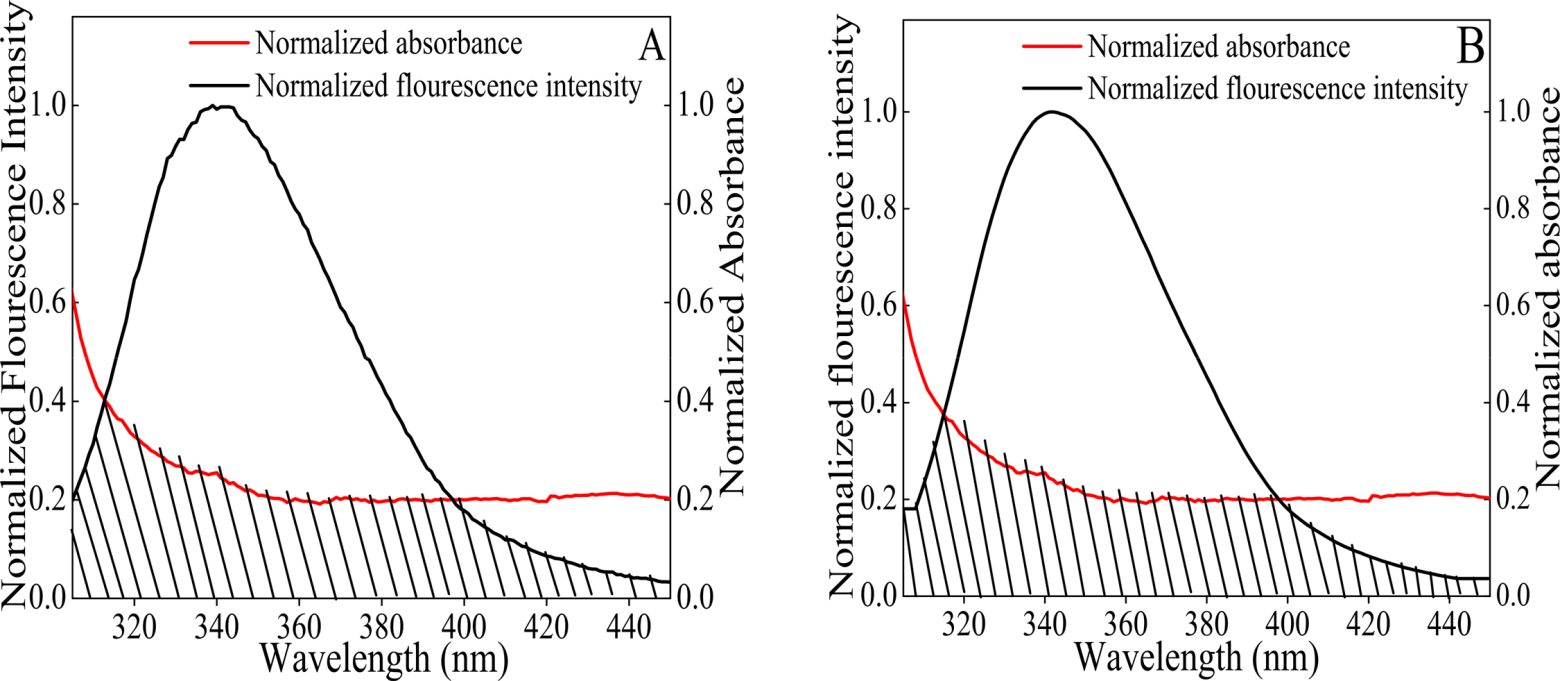
Overlap spectra of normalized absorbance of NDGA and normalized fluorescence intensity of HSA (A) and BSA (B). NDGA and albumins were taken in equimolar concentration (5 μM).

FRET technique is widely used to determine the proximity of drug and spatial distance between a donor and an acceptor during protein-drug interaction. Fig 1 shows that on gradual titration of NDGA, the fluorescence intensity of tryptophan in HSA and BSA decreases. This indicates the transfer of energy from tryptophan to NDGA that results in quenching. The absorption spectrum of NDGA extensively overlaps the emission spectrum of both HSA and BSA as shown in Fig 7A and 7B.If the emission spectrum of donor (HSA and BSA) significantly overlaps with the absorption spectrum of acceptor (NDGA), these donor-acceptor pairs will be considered in Förster distance and the possibility of energy transfer could be ascertained [49]. Therefore, the degree of energy transfer depends upon the area of overlap and the distance between these donor-acceptor molecules (Fig 7A and 7B). Table 3 summarizes the parameters obtained by using the Eqs 5–7. The binding distance (r) obtained for the NDGA-Albumins (HSA & BSA) interactions are 1.43 nm & 1.51 nm, respectively. Further, the R_o_ value is 1.89 nm for both the systems. The value of r and R_o_ is in accordance with the Förster’s non–radiative energy transfer theory [48,50], which states that 0.5R_o_< r<1.5R_o_. This further justified that energy transfer has taken place from HSA and BSA to NDGA that resulted in the quenching of fluorescence intensity.

**Table 3:** FRET parameters obtained from NDGA binding to HSA and BSA.

	J (cm ^3^.M ^-1^)	R_0_ (nm)	r (nm)	*E*_FRET_
**HSA**	2.11 x 10^13^	1.89	1.43	0.71
**BSA**	2.11 x 10^13^	1.89	1.51	0.62

### 3.7. Circular Dichroism (CD) Analysis

**Fig 8:**
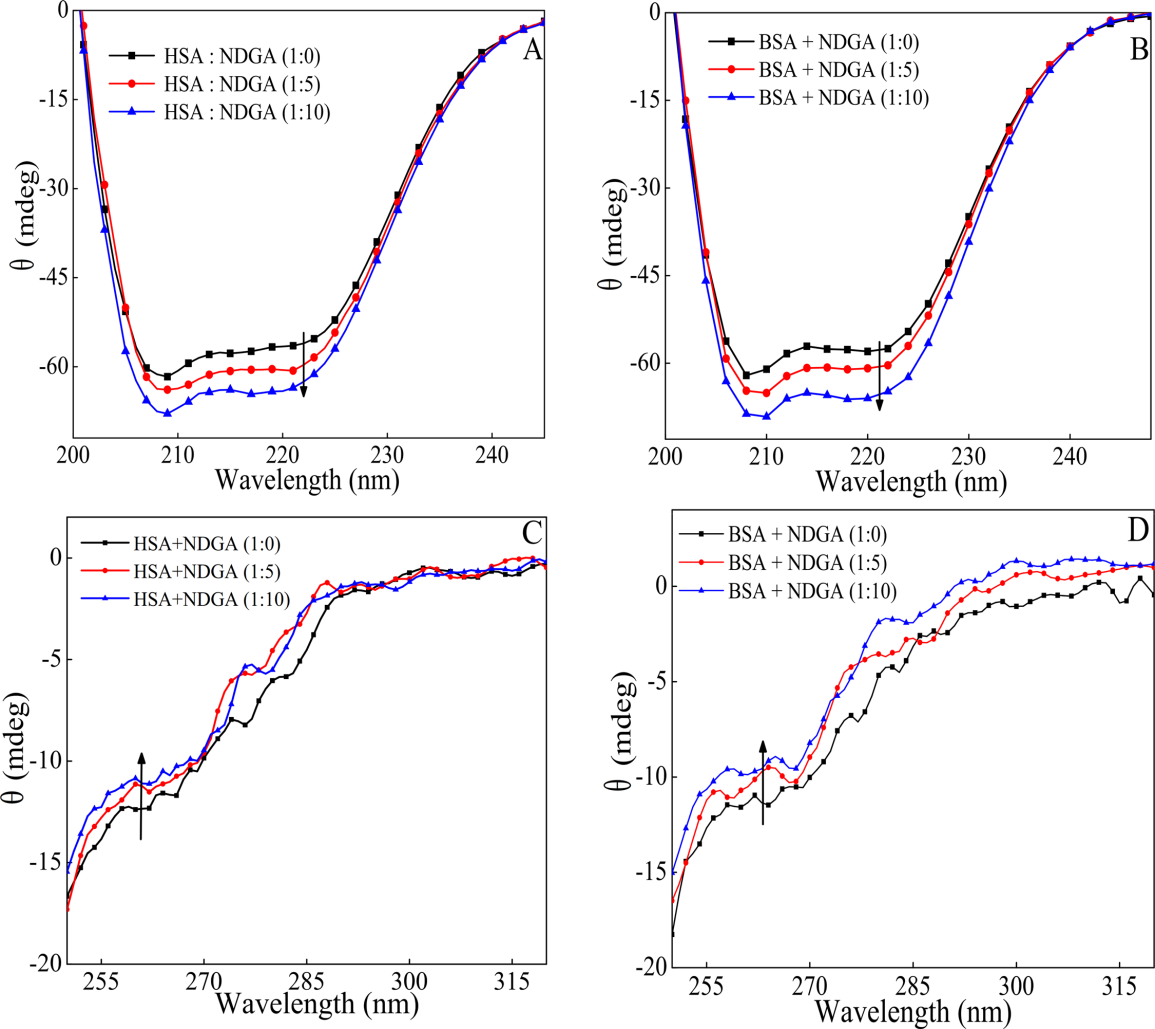
Far-UV (A and B) and near-UV (C and D) CD spectra of HSA and BSA in the presence of 1:0, 1:5 and 1:10 molar ratio of albumins to NDGA.

CD spectroscopy plays an important role in the study of protein-drug interaction as it allows the characterization of secondary as well tertiary structure of the protein upon binding with the drug. Far-UV CD spectra is used to characterize the secondary structure [51] whereas near-UV CD spectra is employed to observe modification in the tertiary structure of protein. Fig 8A and 8B represents the far-UV CD spectra of HSA and BSA at 1:0, 1:5 and 1:10 protein to NDGA molar ratio. In the absence of NDGA, HSA and BSA exhibits two characteristic peak which are around at 222 nm (n→pi* transition) and 208 nm (pi→pi* transition), a characteristic of α-helix [52,53]. The spectra in the Fig 8A and 8B depicts that NDGA interaction with HSA (& BSA) led to a change in secondary structure of albumin resulting into the increment in helical content of protein. Using K_2_D software, the helicity of proteins were calculated. At 1:10 protein to drug molar ratio of NDGA the helicity was found to increase from 55.25±1.05% to 58.35±1.75% in for HSA and from 62.71±1.37% to 65.60±1.28% for BSA. This indicates that NDGA stabilized the secondary structure of HSA and BSA. Fig 8C and 8D shows the near-UV CD spectra of HSA and BSA at 1:0, 1:5 and 1:10 molar ratio of protein to NDGA. Near-UV CD spectra of proteins in the absence of NDGA represents two minima at 262 nm and 268 nm and shoulders at 279 nm and 290 nm, respectively which are attributed to the disulfide bonds and aromatic chromophores [54]. In the presence of NDGA, the near-UV CD spectra of both the proteins showed some changes which signifies tertiary structure alterations.

### 3.8. Dynamic Light Scattering (DLS) Measurements

**Fig 9:**
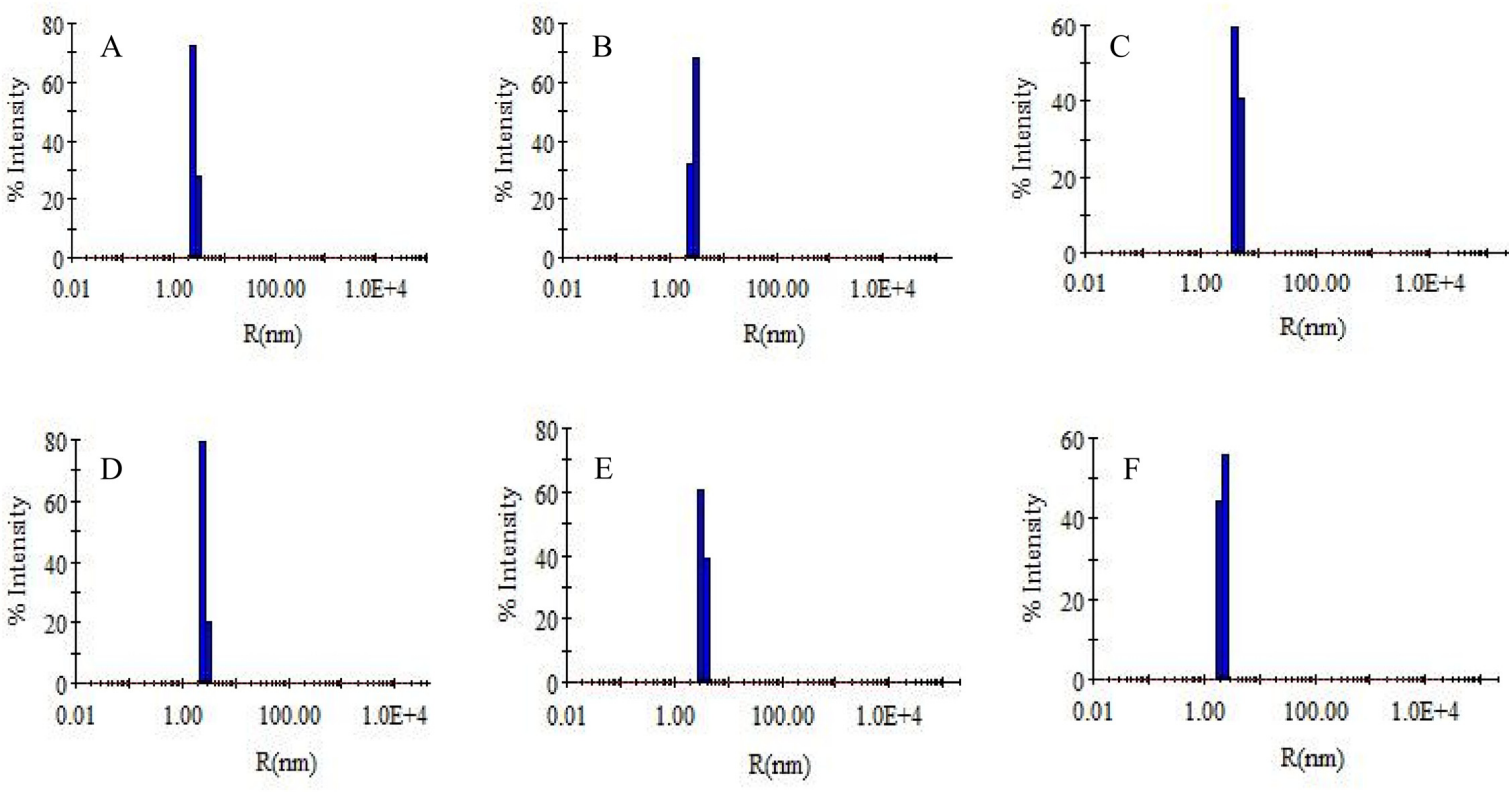
Hydrodynamic radii pattern of HSA and BSA in the absence (A and D) and presence of 1:5 (B and E) and 1:10 molar ratio of albumins to NDGA (C and F).

DLS is used to monitor the change in size of protein molecules [55,56]. Changes in size were monitored by measuring the hydrodynamic radii (*R*_*h*_) of the complex formed *i*.*e*., NDGA-HSA & NDGA-BSA. Fig 9A, 9B and 9C represents the change in *R*_*h*_ values of HSA upon interaction with 1:0, 1:5 and 1:10 molar ratio of protein to NDGA, Fig 9D, 9E and 9F shows the change in *R*_*h*_ values of BSA upon interaction with 1:0, 1:5 and 1:10 molar ratio of protein to NDGA and the obtained DLS parameters *i*.*e*. *R*_*h*_ and polydispersity are summarized in Table 4 [57]. *R*_*h*_ of native HSA and BSA were found to be 3.6 nm & 3.8 nm, respectively which decreased upon interaction with NDGA. Plausibly, it may be due to the interaction of NDGA with albumins in such a manner that the solvent shell surrounding the protein got disrupted causing protein molecule to collapse leading to a decrease in hydrodynamic radii.

**Table 4:** Hydrodynamic radii and polydispersity of HSA and BSA in the absence and presence of NDGA.

Conditions	R_h_	Pd%
**A. HSA**	3.6 ± 0.12	11.0
**B. HSA:NDGA (1:5)**	3.0 ± 0.14	11.9
**C. HSA:NDGA (1:10)**	2.7 ± 0.13	12.5
**D. BSA**	3.8 ± 0.10	10.4
**E. BSA:NDGA (1:5)**	3.4 ± 0.11	11.5
**F. BSA:NDGA (1:10)**	2.8 ± 0.13	11.9

### 3.9. Molecular Docking Studies on NDGA-Albumins (HSA & BSA) Interaction

**Fig 10:**
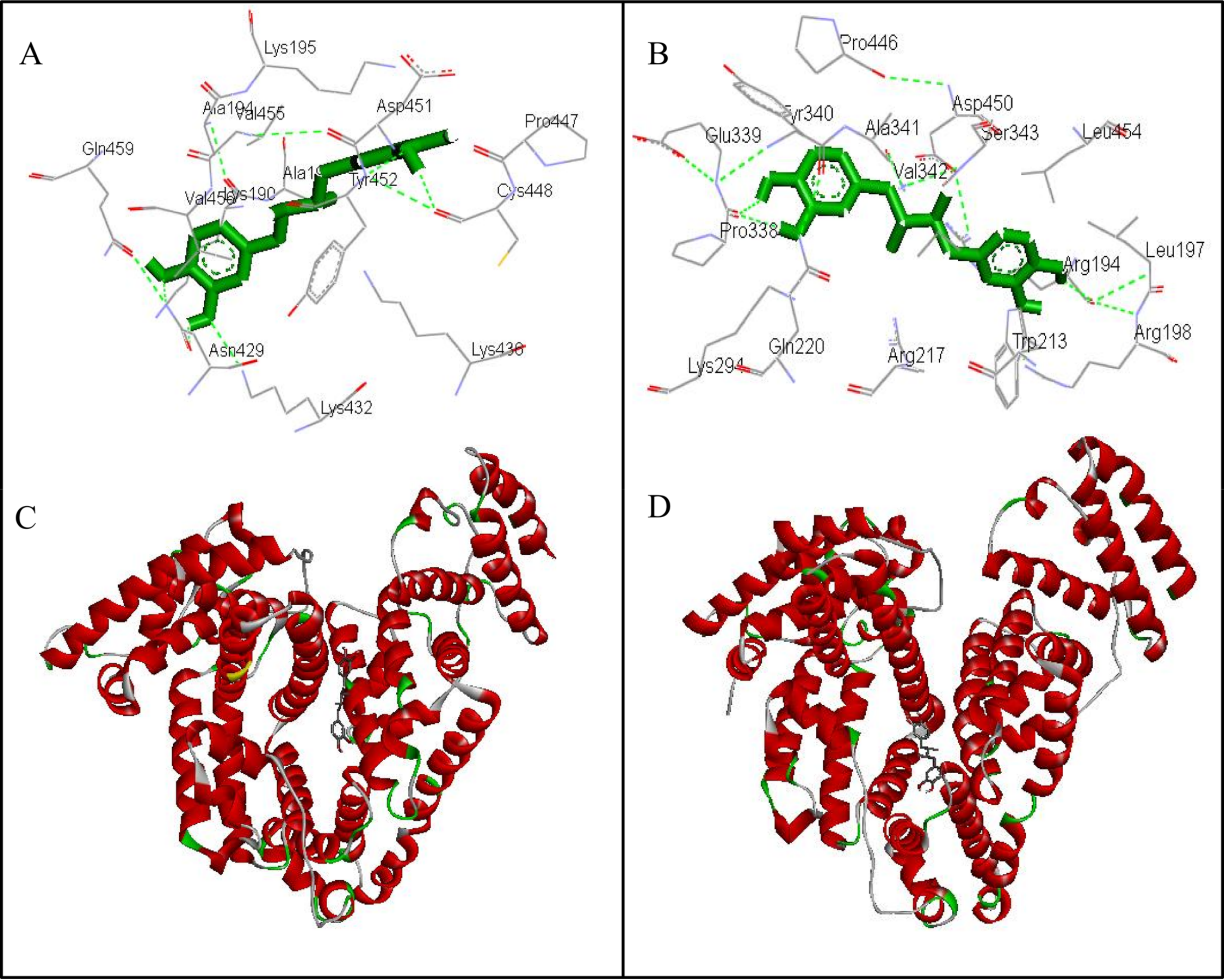
Molecular docking of NDGA and albumins. (A) amino acid residues involved for NDGA interaction at site II of HSA (A) and BSA (B). (C) and (D) Cartoon model representing NDGA as stick while HSA and BSA are represented by ribbon model.

**Fig 11:**
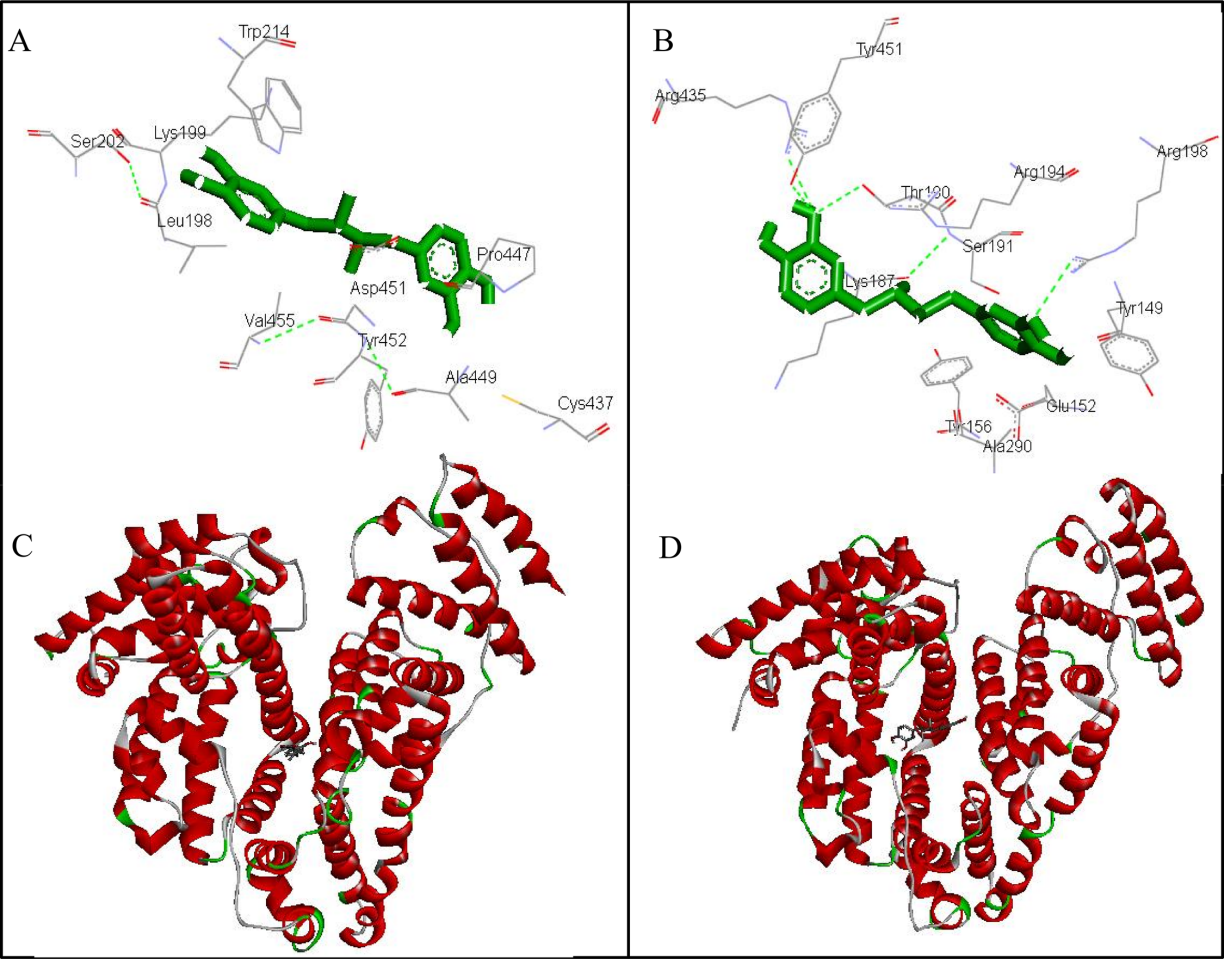
Molecular docking of NDGA and albumins. (A) amino acid residues involved for NDGA interaction at site I of HSA (A) and BSA (B). (C) and (D) Cartoon model representing NDGA as stick while HSA and BSA are represented by ribbon model.

The molecular docking studies were carried out to disclose the binding sites and amino acid residues involved in the interaction of NDGA to the different sites on HSA and BSA. Molecular docking results showed that NDGA primarily interacts with site II but also binds to site I on HSA as well as on BSA [46]. The best energy ranked results and the amino acid residues involved in the interaction are shown in Figs 10 and 11 and summarized in Table 5. As shown in (Fig 10A and 10C) and (Fig 10B and 10D), NDGA positively fits into the hydrophobic compartment close to sub domain IIIA in Sudlow site II of HSA and BSA, with ΔG values of -7.65 kcal.mol^-1^ and -7.12 kcal.mol^-1^, respectively. NDGA interacts hydrophobically with Lys190, Ala191, Ala194, Lys195, Lys432, Lys436, Pro447, Asp451,Val455 and Val456 residues of HSA near site II whereas Asn429, Cys448, Asp451, Tyr452 and Gln459 forms hydrogen bonds with NDGA, as indicated by green dotted lines in Fig 10A. On the contrary Trp213, Arg217, Gln220, Lys294, Tyr340, Ala341, Ser343, Pro446, Asp450 and Leu454 residues present in site II of BSA interacts with NDGA hydrophobically and Arg194, Leu197, Arg198, Pro338, Glu339 and Val342 residues are involved in hydrogen bond formation (Fig 10B), at site II of BSA. Moreover, NDGA also interacts with crucial amino acid residues at site I of HSA and BSA, with ΔG values of -6.26 kcal.mol^-1^and -6.97 kcal.mol^-1^, respectively as shown in (Fig 11A and 11B). The hydrogen bond is formed by Leu198, Ser202, Tyr452 and Val455 residues of Sudlow’s site I in HSA and Thr190, Ser191, Arg198 and Tyr451 residues of Sudlow’s site I in BSA and -OH group of NDGA molecule. Thus, hydrogen bonding and hydrophobic interactions are the major binding forces involved in NDGA and albumin interaction [40].

**Table 5:** Molecular docking parameters obtained from Albumin- NDGA interaction.

	Binding Site	Amino acid residues	Forces involved	ΔG(kcal.mol^-1^)
**HSA**	**Site I**	Lys199,Trp214,Cys437, Pro447,Ala449,Asp451,	Hydrophobic interaction	-6.26
		Leu198,Ser202,Tyr452, Val455,	Hydrogen bond	
	**Site II**	Lys190,Ala191,Ala194, Lys195,Lys432,Lys436, Pro447,Asp451,Val455, Val456	Hydrophobic interaction	-7.65
		Asn429,Cys448,Asp451, Tyr452,Gln459	Hydrogen bond	
**BSA**	**Site I**	Tyr149,Glu152,Tyr156, Lys187,Arg194,Ala290, Arg435	Hydrophobic interaction	-6.97
		Thr190,Ser191,Arg198, Tyr451	Hydrogen bond	
	**Site II**	Trp213,Arg217,Gln220, Lys294,Tyr340,Ala341, Ser343,Pro446,Asp450, Leu454	Hydrophobic interaction	-7.12
		Arg194,Leu197,Arg198, Pro338,Glu339,Val342	Hydrogen bond	

## 4. Conclusion

In the present work we have performed the spectroscopic and molecular docking studies of an antineoplastic and anti-inflammatory drug NDGA with the water soluble model protein i.e. Human and Bovine serum albumins. Results illustrate that the mechanism of fluorescence quenching of HSA and BSA by NDGA is in a static manner and their binding process is spontaneous and enthalpically driven involving both hydrogen bonding and hydrophobic interactions. Synchronous fluorescence spectra show microenvironment change around the tryptophan and also the tyrosine residues. DLS results revealed that NDGA led to the decrement in hydrodynamic radii of albumins causing change in topology of proteins. Far-UV CD spectra indicated the increase in percent alpha helix when complexed with NDGA in case of HSA as well as BSA. Further, molecular docking and fluorescence displacement results indicated that the NDGA molecule actively binds to Sudlow’s site II of albumins (HSA and BSA) and also binds to Sudlow’s site I of both the albumins. Moreover, the values of ΔG calculated from molecular docking are concurrent with those obtained from fluorescence quenching experiment.

Being an effective ROS scavenger, NDGA has been proved to have encouraging relevance in the medication of diseases, like neurological disorders, cardiovascular diseases and cancers, and in the field of tissue engineering. Its medicinal properties have been supported by *in vitro* and *in vivo* experimental studies. In view of the developing appeal for anti-inflammatory and antineoplastic drugs their interaction with major carrier proteins like serum albumins, when used as a treatment drug could be momentous. As the binding of drug with carrier proteins is influential for the transport and metabolism of drug.
