# Correction: A Comprehensive Spectroscopic and Computational Investigation to Probe the Interaction of Antineoplastic Drug Nordihydroguaiaretic Acid with Serum Albumins

**DOI:** 10.1371/journal.pone.0164927

**Published:** 2016-10-13

**Authors:** Saima Nusrat, Mohammad Khursheed Siddiqi, Masihuz Zaman, Nida Zaidi, Mohammad Rehan Ajmal, Parvez Alam, Atiyatul Qadeer, Ali Saber Abdelhameed, Rizwan Hasan Khan

There is an error in the last sentence of the Acknowledgments section. Specifically, the research group number is incorrect. Please see the correct sentence here: The authors would like to extend their sincere appreciation to the Deanship of Scientific Research at King Saud University for its funding this Research Group No. RG-1435-025.

There is an error in the Funding Disclosure statement. The correct Funding Disclosure statement should read: Funding was provided by the Department of biotechnology, Government of India as project BT/PR 13194/BRP/10/742/2009 and DST SR/SO/BB-0018/2011 to RHK; the University Grant Commission, New Delhi, MANF-SRF to SN and MZ; the University Grant Commission, New Delhi, UGC-post doctoral fellowship to NZ; the University Grant Commission, New Delhi, UGC-SRF (NET) fellowship to MRA; the Department of Biotechnology (DBT) DBT-JRF (NET) fellowship to MKS; and the Deanship of Scientific Research at King Saud University for its funding this Research Group No. RG-1435-025. The funders had no role in study design, data collection and analysis, decision to publish, or preparation of the manuscript.
